# Treatment Durability of Limited Fasciectomy versus Percutaneous Needle Fasciotomy for Dupuytren Disease

**DOI:** 10.1097/PRS.0000000000011322

**Published:** 2024-01-30

**Authors:** Bente A. van den Berge, Fatuma M. A. Omar, Paul M. N. Werker, Zhuozhao Zhan, Edwin R. van den Heuvel, Dieuwke C. Broekstra

**Affiliations:** Groningen and Eindhoven, the Netherlands; From the 1 Department of Plastic Surgery, University Medical Center Groningen, University of Groningen; 2 Department of Mathematics and Computer Science, Eindhoven University of Technology.

## Abstract

**Background:**

Patients with Dupuytren disease (DD) are mostly surgically treated by percutaneous needle fasciotomy (PNF) or limited fasciectomy (LF), but data on time intervals to retreatment are lacking. The authors aimed to estimate the risk of retreatment within certain time periods after treatment with PNF and LF.

**Methods:**

The authors used data of participants of a cohort study on the course of DD who were treated only with PNF or LF. The primary outcome measure was time to retreatment of DD. The authors included sex, age at first treatment, and having a first-degree relative with DD as confounders in our analysis. A bivariate gamma frailty model was applied to estimate the risk of retreatment within 1, 3, 5, 10, and 20 years after treatment with PNF and LF.

**Results:**

The time to retreatment was significantly shorter after treatment with PNF than after LF (Wald test statistic, 7.56; *P* < 0.001). The estimated 10-year risk of retreatment for men who underwent their first treatment at a younger age and with a first-degree relative with DD was 97% after PNF and 32% after LF. The estimated 10-year risk for women who underwent their first treatment at an older age without a first-degree relative with DD was 20% after PNF and 6% after LF.

**Conclusions:**

The results show that the patients treated with PNF have a higher risk of retreatment. The results of this study could contribute to individualized information on the treatment durability in the future, which would improve patient counseling about the expected retreatment needs.

**CLINICAL QUESTION/LEVEL OF EVIDENCE:**

Therapeutic, III.

Dupuytren disease (DD) can lead to functional impairment of the hands when flexion deformities of the fingers develop. The ring finger and the little finger are most frequently affected.^1^ Joint contractures can be treated in several ways, but 2 of the most used treatments are limited fasciectomy (LF) and percutaneous needle fasciotomy (PNF).^2^ LF is the standard surgical procedure in which all affected tissue in the operated field is removed without removal of the uninvolved tissue or overlying skin, whereas in PNF the cord is interrupted to relieve the contracture. Both treatments can effectively reduce extension deficits of the finger.^3^ In the short term, PNF results in faster improvement of hand function and fewer major complications compared with LF.^2,3^ In the longer term, recurrence of contractures seems to be more common after PNF than after LF, but these results are based on trials with a limited number of study measurements and follow-up times limited to 5 years.

Recurrence after treatment for DD has been defined in many ways in the past.^4,5^ In 2 previous comparative studies on recurrence after LF and PNF, recurrence was defined as an increase of 20 degrees and 30 degrees in passive extension deficit and total passive extension deficit (TPED), respectively.^6,7^ The advantage of these definitions is that you can speak of “true recurrence” because you objectively measure recontracture and thereby recurrence of the disease. However, to determine recurrence risk in the long-term using this definition, many repeated measurements over a long period are required. Thus, no comparative studies have examined the risk of recurrence in the same finger more than 5 years after treatment, resulting in a knowledge gap in the time to recurrence for these modalities. In addition, recurrence of joint contracture to a certain figure (20 degrees of passive extension deficit or 30 degrees of TPED) does not necessarily mean that a patient chooses for another treatment: some patients may accept their disability considering the pain and recovery time associated with undergoing surgery. Although defining recurrence as retreatment in the same finger implies the risk of missing patients with “true recurrence” refraining from further treatment, this definition would enable us to estimate the recurrence rate in the longer term.

Not only the time to retreatment but also factors that affect this are important to our knowledge about the time interval of DD treatment. Although several risk factors—such as sex, age at first treatment, and genetic predisposition—have been associated with disease recurrence, these factors are not always considered as confounders in studies on recurrence.^7^ Patient-specific estimates on the risk of retreatment could help clinicians to inform patients better and improve their role in patient-centered clinical decision-making. In this observational study, we aim to estimate the risk of retreatment after treatment with PNF and LF.

## PATIENTS AND METHODS

### Study Design and Population

We used data of patients who participated in a prospective cohort study on the course of DD.^8^ For the aim of this study, we included all patients treated for DD with PNF or LF in at least 1 hand. We excluded hands of patients who did not attend any follow-up visits after the first study visit, hands of patients who received a treatment other than PNF or LF, and patients whose treatment date before participation in this study were missing. Ethical approval for this study was obtained from our institutional ethics review board (METc2011/397). All patients provided written informed consent.

### Outcome Measures

Our primary outcome measure was time to retreatment for DD (including time to first treatment) for each ray separately, focusing on the ring and little fingers. In addition, we collected information on potential risk factors for disease retreatment: age at first treatment, sex, and having a first-degree relative (FDR) with DD.

### Procedures

Between 2012 and 2017, all patients were examined every 6 months, and every 12 months since 2018. At the first study visit, patients were asked whether they had been treated for DD in the past and were physically examined for the presence of surgical scars of LF. Patient files and surgical reports from our institution were assessed, and intervention date and type, treatment side, and treated rays were registered. If the patient was treated in another institution, data were requested with the patient’s permission, and if needed, supplemented with information obtained from participants at each study visit. At each following study visit, patients were asked whether they were treated for DD between the previous and the current study measurement. We also monitored the surgery schedules to identify patients planned for surgery. We used the data on treatments and time to retreatment to estimate the risk of retreatment within certain time periods after treatment with PNF and LF by applying a survival model.

### Statistical Analysis

The characteristics of the patients were presented by means and standard deviations for normally distributed, continuous variables. Nonnormally distributed continuous variables and ordinal variables were described by medians with interquartile ranges (IQRs). For dichotomous variables, frequencies and proportions with corresponding confidence intervals were used.

We did not assign patients to separate treatment groups, as most patients underwent different treatment types in the same finger (eg, PNF, PNF, LF; or PNF, LF, LF). Therefore, we included all treated patients as 1 group in our analysis and modeled the time to retreatment after each LF and PNF treatment. We applied a parametric bivariate gamma frailty model^9^ to analyze the time to (re)treatment. The time to retreatment was defined as the time in years between the previous treatment and the next treatment within the same finger, whereas the time to first treatment was defined as the age of the individual at first treatment. Because the number of treatments in the thumb, index, and middle fingers were too low to be included, we focused on retreatment in the ring and little fingers. We analyzed the time to retreatment of these fingers simultaneously because we assume that risk of retreatment of fingers of the same hand are correlated. This was done by assigning a frailty term to the hazard function for the ring and little fingers of each hand separately, which together would form the bivariate gamma distribution with a correlation between the 2 frailties. A frailty term makes the baseline risk patient-specific (and here even finger-specific). These frailty terms also address the heterogeneity between hands because some patients may need more treatments than others. We assumed that treatment for primary and recurrent disease is equally effective.^10^ We reasoned that sex, age at first treatment (standardized), and having an FDR with DD are related to both the exposure (treatment type) and the outcome (retreatment). Therefore, we included these factors as confounders in our model. Patients with missing values in 1 of these variables were excluded from the analysis.

We found that a bivariate gamma frailty model with a lognormal baseline hazard for the time to retreatment fit best according to the log likelihood out of all baseline hazards. (**See Table, Supplemental Digital Content 1**, which shows the log likelihood [*LL*] for different choices of baseline hazard functions for the time to first treatment and the time to retreatment. The combination of a baseline log logistic distributed time to first treatment and baseline log normal distributed time to retreatment gives the highest log likelihood [−991.325]. Thus, this is the combination of baseline hazard distributions that we have used in our analysis, http://links.lww.com/PRS/H102.) Sex (binary), having an FDR (binary), and age at first treatment (standardized continuous) were added to the model to adjust for their potential confounding effect. We compared the effect of PNF, LF, and the confounders on the time to retreatment using Wald test. A two-sided value of *P* < 0.05 was considered significant. All analyses were performed in R v.4.1.3^11^ by creating our own codes (provided on request).

With the estimates from the best fitting model, we estimated the risk of retreatment within 1, 3, 5, 10, and 20 years after PNF or LF. In addition, we plotted the risk of retreatment after treatment with PNF and LF for men and women separately, for patients with and without an FDR with DD, stratified by the age at their first treatment for DD (40, 60, or 80 years old).

## RESULTS

### Population Characteristics

Of 261 participants with DD, 137 were treated for DD in at least 1 finger. Three patients were excluded because their treatment date was unknown, and 3 patients were excluded because they did not attend any follow-up measurement after the baseline visit. One patient was excluded from the analysis because it was unknown whether they had a family history of DD. Furthermore, a total of 30 hands were excluded because they had undergone alternate treatments apart from PNF or LF in the ring and/or little finger. These instances were distributed among 26 separate patients, resulting in the exclusion of 13 participants. The other hand of the remaining 13 participants met the criteria, so these participants were not excluded. After these exclusions, 117 patients were included in the analyses.

Of 117 patients, 82 (70%) were men and 35 (30%) were women. Eighty-nine patients (76%) did not undergo repeated treatment in any finger, whereas 28 patients (24%) were treated more than once in at least 1 digit. A summary of the baseline characteristics is represented in Table 1. Together, these patients underwent 247 treatments on the ring and little fingers. It is important to note that some of ring and little fingers were treated simultaneously. A total number of 157 (64%) ring and little fingers treated with LF and 90 (36%) fingers treated with PNF were included in the analysis. All patients who received a treatment other than LF or PNF were excluded. However, to provide insight into all treatment combinations that occurred in our study population, we have included an overview of these. (**See Table, Supplemental Digital Content 2**, which shows the order and combinations of treatments in which each finger has been treated, http://links.lww.com/PRS/H103.)

**Table 1.** Baseline Characteristics of All 117 Patients^a^

Characteristic	Patients Treated Once (%)	Patients with Repeated Treatment (%)	Total (%)
No.	89 (76)	28 (24)	117 (100)
Mean age at first treatment ± SD	64.4 ± 8.7	62.8 ± 9.3	64.0 ± 8.9
Male sex	59 (66)	23 (82)	82 (70)
First-degree relative, yes	47 (53)	14 (50)	61 (52)
Follow-up, yr			
Median	7.1	7.8	7.2
IQR	3.7–11.3	5.9–11.1	3.8–11.3
Missing	1	0	1

a All reported values (except follow-up time) were measured at the time of inclusion. If missingness was not reported, there were no missing values for that variable. Follow-up time represents the number of years between initial treatment and the end of the study period.

### Time to Retreatment

Of 95 treated ring fingers, 28 were primarily treated with PNF, and 67 with LF. Ten (36%) ring fingers were retreated after primary treatment with PNF after a median time to retreatment or censoring (ie, end of follow-up) of 3.3 years (IQR, 2.0 to 5.2 years). Four (6%) ring fingers underwent retreatment after primary treatment with LF, after a follow-up of 8.0 years (IQR, 4.3 to 11.7 years).

Of 100 treated little fingers, PNF was the first treatment in 32 fingers, and LF was the first treatment in 68 fingers. Thirteen little fingers (41%) were retreated after primary treatment with PNF after a follow-up of 3.1 years (IQR, 2.1 to 5.0 years). Eight (12%) were retreated after primary treatment with LF after a follow-up of 7.1 years (IQR, 3.9 to 10.6 years). The time to retreatment after the first, second, third, or fourth treatment of the ring and little fingers with PNF or LF is reported in Table 2.

**Table 2.** Time to Retreatment after the First, Second, Third, and Fourth Treatment in the Ring and Little Fingers Treated with PNF and LF^a^

	PNF		LF	
	Ring Finger	Little Finger	Ring Finger	Little Finger
First treatment, n	28	32	67	68
Retreatment after first treatment, n	10	13	4	8
Years to retreatment, median (IQR)	3.3 (1.7–4.5)	3.4 (2.1–4.8)	4.0 (3.0–7.8)	5.5 (3.5–7.2)
Years to censoring, median (IQR)	3.2 (2.1–5.6)	3.0 (2.2–6.3)	8.0 (4.7–11.7)	7.8 (4.1–11.0)
Second treatment, n	8	9	6	12
Retreatment after second treatment, n	3	5	0	2
Years to retreatment, median (IQR)	2.2 (1.9–2.2)	1.9 (1.9–2.5)	—	5.8 (5.3–6.3)
Years to censoring, median (IQR)	3.5 (3.1–4.0)	5.1 (3.9–6.7)	6.5 (5.5–7.2)	7.0 (4.3–7.7)
Third treatment, n	3	5	0	2
Retreatment after third treatment, n	1	4	0	1
Years to retreatment, median (IQR)	0.8 (—)	2.1 (1.7–2.8)	—	3.6 (—)
Years to censoring, median (IQR)	2.4 (2.1–2.4)	3.9 (—)	—	11.5 (—)
Fourth treatment, n	1	3	0	2
Retreatment after fourth treatment, n	1	0	0	0
Years to retreatment, median (IQR)	1.0 (—)	—	—	—
Years to censoring, median (IQR)	—	2.7 (1.7–2.8)	—	4.5 (3.1–5.9)
Fifth treatment, n	1	0	0	0
Retreatment after fifth treatment, n	—	—	—	—
Years to retreatment, median (IQR)	—	—	—	—
Years to censoring, median (IQR)	0.04 (—)	—	—	—

a A third, fourth, and fifth treatment in some cases took place in only 1 finger. Therefore, no IQR was reported for these fingers.

**Fig. 1.**
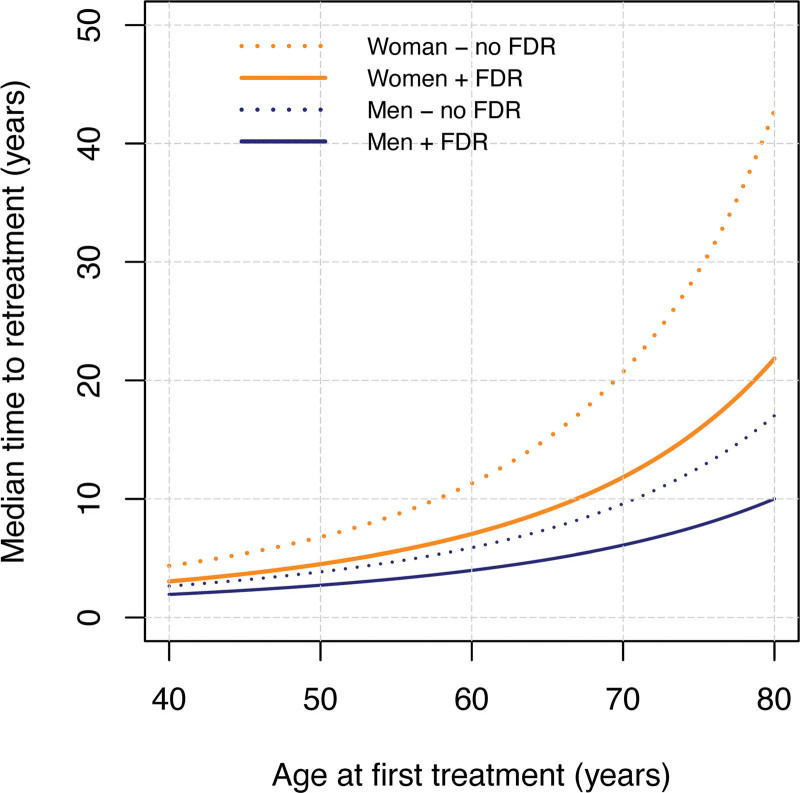
A plot of the estimated median time to retreatment after PNF.

The time to retreatment was significantly shorter after PNF than after LF (Wald test statistic, 7.56; *P* < 0.001). The estimated time to retreatment after PNF for men and women, with and without an FDR is presented in Figure 1. The number of events in patients treated with LF was too small to reliably estimate the time to retreatment after LF.

### Heterogeneity and Correlation

Our statistical model included frailty terms that address the heterogeneity between the hands of 1 patient and the correlation between the risk of the ring and little fingers. The heterogeneity was 0.33 (SE, 0.21; *P* = 0.11; test statistic, 1.58), meaning that the heterogeneity between hands was substantial. This can be explained by the fact that there are patients in the study who were not treated in the ring and/or little finger and that were patients treated multiple times in both hands. The Pearson correlation coefficient between the risk of the ring and little fingers was 0.12 (SE, 0.36; *P* = 0.74; test statistic, 0.34).

### Risk of Retreatment after PNF

**Fig. 2.**
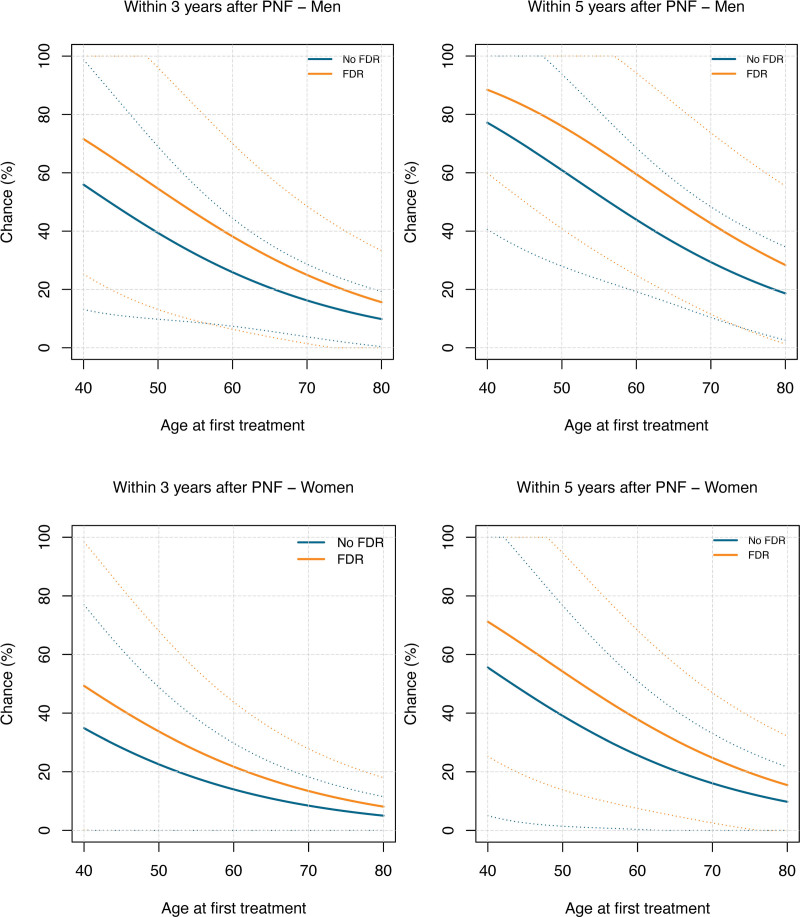
Plots of the risk of retreatment for men (*above*) and women (*below*) within 3 and 5 years after treatment with PNF. The *x* axis represents age at first treatment in years, and the *y* axis represents the chance of retreatment.

Men aged 40 years at first treatment who had an FDR with DD had an estimated risk of retreatment of 71% within 3 years and 88% within 5 years after PNF, compared with 55% and 77% for men without an FDR. Women aged 40 years at first treatment with an FDR with DD had an estimated risk of 49% within 3 years and 71% within 5 years after PNF, compared with 34% and 55% for women without an FDR (Fig. 2). All estimated risks after 1, 3, 5, 10, and 20 years stratified by age and FDR are shown. (**See Table, Supplemental Digital Content 3**, which shows [*above*] the estimated risk of retreatment within 1, 3, 5, 10, and 20 years for men and women without an FDR with DD, treated with PNF or LF. [*Below*] Estimated risk of retreatment within 1, 3, 5, 10, and 20 years for men and women with an FDR with DD, treated with PNF or LF, http://links.lww.com/PRS/H104.)

**Fig. 3.**
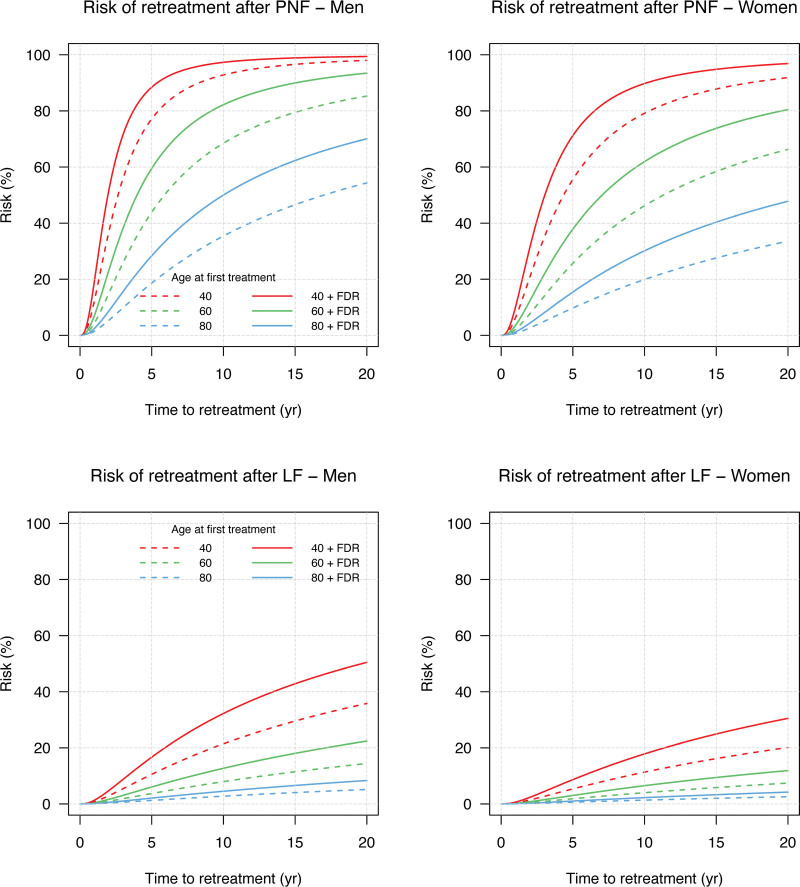
Plots of the risk of retreatment after PNF and LF. The *x* axis represents the time to retreatment in years, and the *y* axis represents the risk of retreatment (%).

We estimated the risk of retreatment also for longer periods. Men who have an FDR with DD and are relatively young at their first treatment are at high risk of retreatment after PNF. For example, a 40-year-old man with an FDR with DD had an estimated risk of retreatment of 97% within 10 years and 99% within 20 years. A woman with the same characteristics had an estimated risk of 89% within 10 years and 97% within 20 years (Fig. 3). A younger age at first treatment was associated with a higher the risk of retreatment. However, the effect of sex and having an FDR were not statistically significant. (**See Table, Supplemental Digital Content 4**, shows the maximum likelihood estimates and 95% asymptotic confidence interval. Effect of the confounders on the time to first treatment and the time to retreatment using the Wald test, http://links.lww.com/PRS/H105.)

### Risk of Retreatment after LF

**Fig. 4.**
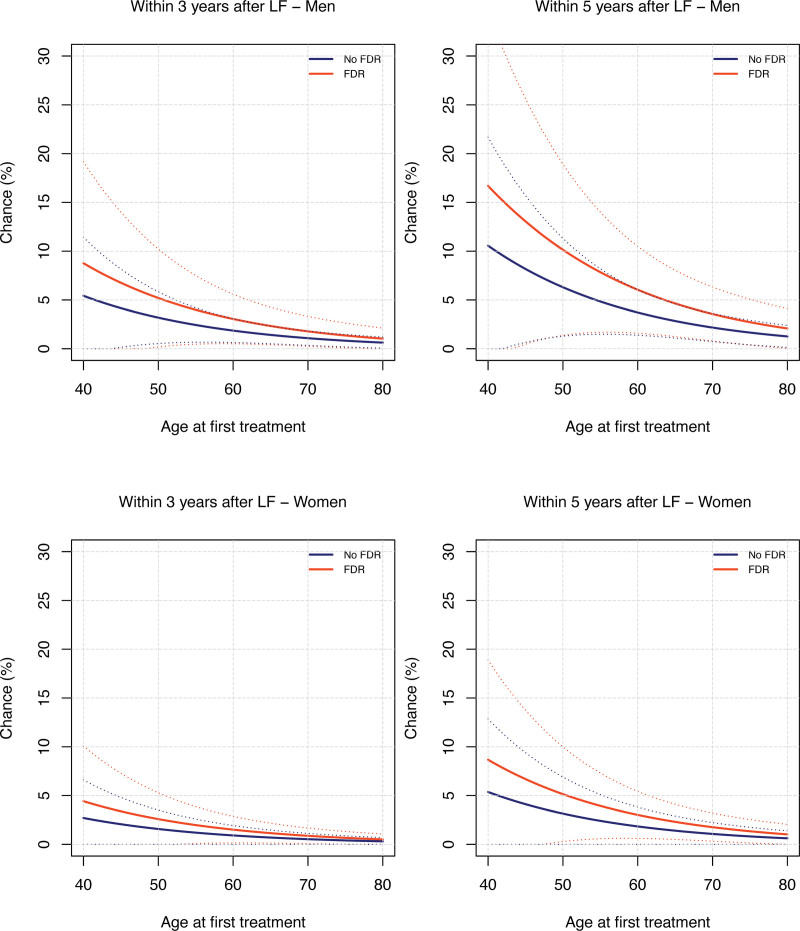
Plots of the risk of retreatment for men (*above*) and women (*below*) within 3 and 5 years after treatment with LF. Note that the scale on the *y* axis (0% to 30%) is different from the scale used in Figure 3 (0% to 100%).

Men aged 40 years at first treatment who had an FDR with DD had an estimated risk of retreatment of 9% within 3 years and 16% within 5 years after LF, compared with 5% and 10% for men without an FDR. Women aged 40 years at first treatment with an FDR with DD had an estimated risk of 4% within 3 years and 8% risk within 5 years after LF, compared with 3% and 5% for women without an FDR (Fig. 4). All estimated risks after 1, 3, 5, 10, and 20 years stratified by age and FDR are shown (**see Table, Supplemental Digital Content 3**, http://links.lww.com/PRS/H104).

Again, men who have undergone treatment at a younger age and who have an FDR with DD are at high risk of retreatment after LF in the longer term. A 40-year-old man with an FDR with DD has 32% risk of retreatment within 10 years, and 50% within 20 years. A woman with the same characteristics has 18% risk of retreatment after 10 years and 30% after 20 years (Fig. 3).

## DISCUSSION

In this study, we present the estimated risk of retreatment for DD. Our results show that the time to retreatment was significantly shorter after treatment with PNF than after treatment with LF. The estimated 10-year risk was highest for men initially treated at 40 with an FDR with DD, being 97% for PNF and 32% for LF. The estimated 10-year risk was lowest for women initially treated at 80 without an FDR with DD, being 20% for PNF and 1% for LF.

After primary treatment with PNF, 38% of the fingers in our study were retreated after a median time to retreatment or censoring of 3.2 years, compared with 9% of fingers treated with LF after 7.5 years. In a previous comparative study with a follow-up period of 1 year, the retreatment rate was 8% in fingers treated with LF and 15% in fingers treated with PNF.^7^ In another study, the 10-year risk of retreatment in the same hand was 19.5% after primary LF and 33.7% after primary PNF.^2^ These findings are in concordance with ours, showing that the time to retreatment in the same finger after LF is significantly longer compared with PNF. Although this study had a significantly larger sample size than our study, retreatment was defined as a first retreatment in the same hand. As a result, an operation of another finger was also regarded as a retreatment. Because we defined retreatment as another treatment in the same finger, our findings give a more accurate estimation of retreatment and further expand current knowledge on retreatment.

Because of the limited follow-up time of previous comparative studies, little is known about the risk of recurrence in the longer term. We therefore estimated the risk of retreatment between 1 and 20 years after initial treatment. Because several risk factors have been associated with an aggressive disease course—also known as the Dupuytren diathesis^12^—and disease recurrence,^13^ we included age at first treatment, sex, and family history (having an FDR with DD) as confounders in our model. In our analysis, the effect of age at first treatment was statistically significant, but the effect of the other confounders was not. Therefore, we cannot confirm or affirm that these factors should be part of the diathesis. It is, however, likely that our sample size was too limited to demonstrate a significant effect. We have therefore decided to show the results stratified for age, sex, and family history. By means of this, physicians can provide patients a better individual estimate of the risk of retreatment in the future, which can help patients to make a well-informed decision about their treatment choice.

When choosing the most suitable treatment option with a patient, not only the treatment-free period but also other important outcomes (eg, efficacy, complications, recovery period, return to work/leisure activities, and patient-reported outcomes) should be considered. The efficacy of PNF and LF are comparable in mildly and moderately affected digits (TPED up to 90 degrees), but PNF is less invasive and associated with a shorter recovery time.^3,14^ In addition, PNF was found to be effective in reducing extension deficits of recurrent disease, so it can be applied repeatedly.^15^ For patients who benefit from a short recovery time or for older patients—who have been shown to have a lower risk of retreatment than younger patients^16^—PNF may be a suitable choice.

The main strengths of our study are the longitudinal study design and our statistical methodology. Because we used longitudinally collected data obtained from regular study visits, we could perform our analyses on reliable data and control for potential confounders. Many studies on the durability of DD treatment are descriptive, self-controlled studies in which medical records are used.^17,18^ In these studies, data of treatments elsewhere have not been included. Consequently, the durability in these studies could be overestimated. Second, we analyzed our data using a parametric bivariate gamma frailty model. This enabled us to make a first estimate of the risk of retreatment in the shorter and longer term. Such information is of great added value for informed shared decision-making of the treatment of DD. In other studies, recurrence was analyzed with logistic regression analysis or *t* tests.^6,13,19^ These methods are suitable for estimating the difference in recurrence after a certain number of years, but the time to recurrence cannot be considered. With our survival model, we could compare time to retreatment and adjust for correlation between fingers within 1 hand, which was moderate (Pearson correlation coefficient, 0.12). This indicates that the risk of treatment between 2 fingers of the same hand is slightly related. This could be explained by factors being shared by the fingers, such as the genetic risk of that patient,^16^ but it is also possible that a surgeon decides to also treat another, (less) affected finger simultaneously because that is more convenient than 2 separate operations.

Our study has limitations. Our data contain more than 1 observation per patient, which means that these observations may be correlated because some characteristics, such as genetic risk, are the same for all fingers belonging to the same patient. Unfortunately, we could not account for the potential correlation between the hands belonging to 1 patient, because this would have led to an increased uncertainty of the parameter estimates in our relatively small sample size. For the same reason, we could not account for potential differences in time to retreatment after primary versus recurrent disease. Second, including preoperative disease severity would have been preferable. Unfortunately, this information was unavailable. It has, however, been reported that PNF and LF are both effective in treating primary and recurrent joint contractures.^10,15,20^ In that light, including preoperative deformity would have little effect on our risk estimates. Third, application of the Delphi-definition (>20 degrees of contracture recurrence in any treated joint at 1-year compared with 6 weeks after treatment)^21^ would have led to a better comparison of our results with others,^4^ but this was unattainable because we examined our patients at regular intervals. However, a joint contracture of greater than 20 degrees does not necessarily implicate serious functional complaints, whereas the risk of retreatment has high clinical relevance for all patients. In addition, a maximal measurement error in a ray of 15.2 degrees must be considered.^22^ As a result, patients can be wrongly classified as having recurrence—and vice versa—when applying the Delphi definition. Retreatment is a more reliable outcome measure, which underpins the robustness of our results. Lastly, we would have preferred to use age of onset instead of age at first treatment, but during subsequent study visits we noticed that age of onset was highly subject to recall bias, as many patients could not accurately estimate their age of onset.

In this study, we estimated the risk of recurrence after treatment for DD for different periods. These risks were estimated highest for younger men treated with PNF with familial predisposition and lowest for older women treated with LF without familial predisposition. Although this can be seen as a disadvantage of PNF, it is less invasive, has faster recovery, is less expensive, and often can be repeated, leading to very similar results as the primary treatment.^15^ Individualized information on the treatment durability could improve patient counseling about the expected treatment results in the longer term. The results of this study directly contribute to this, by providing the estimated risk of retreatment after PNF and LF, for different patient characteristics.

**CODING PERSPECTIVE**

Coding perspective provided by Jeff Kozlow, MD, MS, is intended to provide coding guidance.

26040 Fasciotomy, palmar (eg, Dupuytren contracture); percutaneous
26045 Fasciotomy, palmar (eg, Dupuytren contracture); open
26121 Fasciectomy, palm only, with or without Z-plasty, other local tissue rearrangement, or skin grafting (includes obtaining graft)
26123 Fasciectomy, partial palmar with release of single digits, including proximal interphalangeal joint, with or without Z-plasty, other local tissue rearrangement, or skin grafting (includes obtaining graft)
+26125 Fasciectomy, partial palmar with release of single digits, including proximal interphalangeal joint, with or without Z-plasty, other local tissue rearrangement, or skin grafting (includes obtaining graft); each additional digit (list separately in addition to code for primary procedure)

- A percutaneous fasciotomy is reported with code 26040 (fasciotomy, palmar [eg, Dupuytren contracture]; percutaneous).
- An open fasciotomy is reported with reported with code 26045 (fasciotomy, palmar [eg, Dupuytren contracture]; open).
- Procedures in which the diseased fascia is excised (ie, fasciectomy) are reported with code 26121 or with code 26123 and/or add-on code 26125, depending on the extent of the procedure and the number of digits released.
  - As noted in the code descriptors, codes 26121, 26123, and +26125 all include local tissue rearrangements and/or skin grafts that might be performed after the release procedure. This has been reflected in the work relative value unit and reimbursement calculations for the “typical patient” undergoing these procedures across a practice.
  - Code 26123 includes fasciectomy of the palm and 1 digit. Additional digits are then reported with add-on code +26125.
- All of these codes have a 90-day global period that includes all related care and subsequent follow-up visits during that period.
- In the Medicare fee schedule, pricing is the same for both a facility (ie, in an operating room) and a nonfacility (ie, in your clinic) for all of these codes. Unlike some codes that have increased Medicare physician reimbursement when performed in the nonfacility due to the additional cost of supplies and other expenses to the practice, these codes do not include these costs when reimbursing the physician, nor are the additional costs reported separately. Individual insurers may have differential pricing based on whether the procedure is performed in clinic versus an operating room to account for the practice expense costs to the provider. For providers who perform these procedures more frequently in their clinic under local anesthesia, this may be a consideration in your contract negotiations with insurers.
- Codes 26040, 26045, 26121, and 26123 have a medically unlikely edit (MUE) of 1, which means that they can only be reported once per session. They also have an MUE adjudication indicator of 2, which means that it is an absolute rule and cannot be overridden by modifiers when reporting to the Centers for Medicare and Medicaid Services (and other insurers who follow National Correct Coding Initiative guidelines). However, the Centers for Medicare and Medicaid Services fee schedule does allow for reporting bilateral procedures (–50 or –RT/–LT), with the standard 50% reduction in reimbursement on the second side.
  - Add-on code +26125 is used to report additional digit release in conjunction with code 26123 for the palm and first digit. This code has an MUE of 4, as the procedure could theoretically be performed on all 5 digits. Use of the FA to F9 modifiers is encouraged to expedite claims.
- Codes 26040 and 26045 also have a pair-to-pair edit that does not allow the codes to be reported together for the same hand.

**CODING PRINCIPLE:** Fasciotomy procedures in the hand for Dupuytren disease are reported with code 26040 or 26045, depending on whether a percutaneous or open approach is performed. More extensive procedures, including fasciectomies, are reported with codes 26121, 26123, and +26125.

**Disclosure:** Jeffrey Kozlow, MD, MS, has no financial disclosures to report. He serves as the American Society of Plastic Surgeons co-advisor to the American Medical Association’s CPT Editorial Panel and Relative Value Scale Update Committee.

## DISCLOSURE

Dr. Werker was a member of a safety and efficacy review board and is a data management committee member for Fidia Ltd. (Milan, Italy). Dr. Werker is a member of the scientific advisory board of the International Dupuytren Society. Drs. Werker and Broekstra are both members of the scientific advisory board of the Dutch Dupuytren Society. This is not related to the submitted work. The remaining authors declare no potential conflicts of interest with respect to the research, authorship, and/or publication of this article.

## ACKNOWLEDGMENT

This research was partly funded by the C&W de Boer foundation. The funding bodies had no influence on the design, conduct, or analyses of this study.

## Supplementary Material


