# Supplementary material for: Treatment Durability of Limited Fasciectomy versus Percutaneous Needle Fasciotomy for Dupuytren Disease
Source: Plast Reconstr Surg. 2024 Jan 30;154(5):928–38. doi: 10.1097/PRS.0000000000011322 (PMC11512621; doi:10.1097/PRS.0000000000011322)
Supplement: Supplementary file 1 [file prs-154-0928e-s001.pdf]

Table, Supplemental Digital Content 1. Loglikelihood (LL) for different choices of baseline hazard functions for the time to first treatment and the time to retreatment.

Hazard first	Hazard retreatment	Loglikelihood	Hazard first	Hazard retreatment	Loglikelihood
Weibull	Weibull	-994.995	Loglogistic	Weibull	-994.829
Weibull	Lognormal	-992.054	Loglogistic	Lognormal	-991.325
Weibull	Loglogistic	-992.874	Loglogistic	Loglogistic	-992.419
Weibull	Gompertz	-1033.91	Loglogistic	Gompertz	-1034.991
Lognormal	Weibull	-999.061	Gompertz	Weibull	-
Lognormal	Lognormal	-995.253	Gompertz	Lognormal	-991.935
Lognormal	Loglogistic	-996.478	Gompertz	Loglogistic	-
Lognormal	Gompertz	-1039.682	Gompertz	Gompertz	-

The combination of a baseline loglogistic distributed time to first treatment and baseline lognormal distributed time to retreatment gives the highest log-likelihood (-991.325). Hence, this is the combination of baseline hazard distributions that we have used in our analysis. Because the number of parameters in each of these models is equal, we could compare the log-likelihood of the models.
