# Supplementary material for: Treatment Durability of Limited Fasciectomy versus Percutaneous Needle Fasciotomy for Dupuytren Disease
Source: Plast Reconstr Surg. 2024 Jan 30;154(5):928–38. doi: 10.1097/PRS.0000000000011322 (PMC11512621; doi:10.1097/PRS.0000000000011322)
Supplement: Supplementary file 2 [file prs-154-0928e-s002.pdf]

Table, Supplemental Digital Content 2. Order and combination of treatments in which each finger has been treated in our cohort.

**Treatment**	**Thumb**	**Index**	**Middle**	**Ring**	**Little**	**Total**
None	158	159	134	60	57	568
PNF	3	1	5	17	19	45
PNF-PNF	0	0	0	6	4	10
PNF-LF	1	1	0	2	5	9
PNF-PNF-PNF	0	0	0	4	2	6
PNF-PNF-PNF-PNF	0	0	0	0	3	3
PNF-PNF-PNF-LF	0	0	0	0	1	1
PNF-PNF-PNF-PNF-PNF	0	0	0	1	0	1
PNF-LF-LF-DF	0	0	0	1	0	1
PNF-LF-LF-LF	0	0	1	0	0	1
DF	0	2	1	0	1	4
LF	7	8	23	65	61	164
LF-PNF	0	0	0	1	0	1
LF-DF	0	0	0	1	2	3
LF-LF	1	0	5	6	5	17
LF-CCH	0	0	0	0	1	1
LF-LF-LF	0	0	0	1	1	2
LF-LF-DF	0	0	0	1	1	2
LF-LF-LF-LF	0	1	0	0	1	2
LF-LF-LF-AMP	0	0	0	0	1	1
LF-DF-SI-SI	0	0	0	0	1	1
LF-LF-CCH-CCH-LF	0	0	0	0	1	1
LF-DF-LF-LF-LF	0	0	0	0	1	1
CCH	0	0	1	2	0	3
CCH-LF	1	0	0	0	1	2
SI	0	0	0	1	0	1
RT	0	0	0	1	0	1
TR-PNF	0	0	1	0	0	1
a-TNF/P	0	0	1	0	1	2
a-TNF/P-PNF-PNF	0	0	0	1	0	1
a-TNF/P-PNF-PNF-PNF	0	0	0	0	1	1

PNF = percutaneous needle fasciotomy; DF = dermofasciectomy;LF = limited fasciectomy; CCH = collagenase clostridium histolyticum. SI = steroid injection; RT = radiotherapy; AMP = amputation; TR = traumatic rupture; a-TNF = anti-tumor necrosis factor injection; P = placebo. NB: all patients with other treatments that PNF or LF have been excluded.
