# Supplementary material for: Treatment Durability of Limited Fasciectomy versus Percutaneous Needle Fasciotomy for Dupuytren Disease
Source: Plast Reconstr Surg. 2024 Jan 30;154(5):928–38. doi: 10.1097/PRS.0000000000011322 (PMC11512621; doi:10.1097/PRS.0000000000011322)
Supplement: Supplementary file 3 [file prs-154-0928e-s003.pdf]

Table, Supplemental Digital Content 3a. Estimated risk of retreatment within 1, 3, 5, 10 and 20 years for men and women without a first degree relative (FDR) with DD, treated with PNF or LF

			Women – no FDR		Men – no FDR	
**Treatment**	**Within**	**Age at first treatment**	**Risk,%(SE)**	**95% CI**	**Risk,%(SE)**	**95% CI**
PNF	1 year	40	6% (4)	(0, 15)	12% (6)	(0, 25)
		50	4% (2)	(0, 8)	7% (3)	(1, 14)
		60	2% (1)	(0, 5)	4% (2)	(1, 8)
		70	1% (1)	(0, 3)	3% (1)	(0, 5)
		80	1% (1)	(0, 2)	2% (1)	(0, 3)
	3 years	40	34% (19)	(0, 71)	55% (17)	(23, 88)
		50	22% (11)	(0, 45)	39% (10)	(20, 59)
		60	14% (7)	(1, 27)	26% (5)	(15, 37)
		70	9% (4)	(0, 17)	17% (4)	(8, 25)
		80	5% (3)	(0, 11)	10% (4)	(2, 18)
	5 years	40	55% (23)	(1, 100)	77% (15)	(47, 100)
		50	39% (17)	(6, 72)	61% (12)	(38, 84)
		60	26% (11)	(4, 48)	44% (7)	(29, 59)
		70	16% (8)	(1, 31)	30% (7)	(17, 43)
		80	10% (6)	(0, 21)	19% (7)	(5, 33)
	10 years	40	79% (20)	(40, 100)	93% (9)	(76, 100)
		50	63%% (19)	(26, 100)	83% (10)	(64, 100)
		60	46% (16)	(16, 77)	69% (9)	(52, 86)
		70	32% (13)	(7, 56)	52% (9)	(34, 70)
		80	20% (10)	(0, 41)	36% (11)	(14, 58)
	20 years	40	92% (12)	(69, 100)	98% (4)	(91, 100)
		50	81% (15)	(52, 100)	94% (6)	(82, 100)
		60	66% (16)	(35, 98)	85% (7)	(71, 100)
		70	50% (15)	(19, 80)	72% (9)	(54, 90)
		80	34% (15)	(5, 63)	55% (13)	(29, 82)
LF	1 year	40	0% (0)	(0, 1)	1% (1)	(0, 2)
		50	0% (0)	(0, 1)	0% (0)	(0, 1)
		60	0% (0)	(0, 1)	0% (0)	(0, 1)
		70	0% (0)	(0, 0)	0% (0)	(0, 0)
		80	0% (0)	(0, 0)	0% (0)	(0, 0)
	3 years	40	3% (2)	(0, 6)	5% (3)	(0, 10)
		50	2% (1)	(0, 3)	3% (1)	(1, 6)
		60	1% (1)	(0, 2)	2% (1)	(0, 3)
		70	1% (0)	(0, 1)	1% (1)	(0, 2)
		80	0% (0)	(0, 1)	1% (0)	(0, 1)
	5 years	40	5% (4)	(0, 12)	10% (5)	(1, 20)
		50	3% (2)	(0, 7)	6% (2)	(2, 11)
		60	2% (1)	(0, 4)	4% (1)	(1, 6)
		70	2% (1)	(0, 2)	2% (1)	(0, 4)
		80	1% (0)	(0, 2)	1% (1)	(0, 3)
	10 years	40	11% (7)	(0, 25)	21% (9)	(4, 38)
		50	7% (4)	(0, 14)	13% (5)	(4, 22)
		60	4% (2)	(1, 8)	8% (3)	(3, 13)
		70	2% (1)	(1, 5)	5% (2)	(1, 9)
		80	1% (1)	(0, 3)	3% (2)	(0, 6)
	20 years	40	20% (11)	(0, 42)	35% (13)	(10, 61)
		50	12% (6)	(0, 25)	23% (7)	(9, 37)
		60	7% (4)	(0, 15)	14% (5)	(5, 24)
		70	4% (3)	(0, 9)	9% (4)	(2, 16)
		80	3% (2)	(0, 6)	5% (3)	(0, 11)

PNF = percutaneous needle fasciotomy; LF = limited fasciectomy; FDR = first degree relative

Table, Supplemental Digital Content 3b. Estimated risk of retreatment within 1, 3, 5, 10 and 20 years for men and women with a first degree relative% (FDR) with DD, treated with PNF or LF

			Women – with FDR		Men – with FDR	
**Treatment**	**Within**	**Age at first treatment**	**Risk,%(SE)**	**95% CI**	**Risk,%(SE)**	**95% CI**
PNF	1 year	40	10% (7)	(0, 24)	19% (10)	(0, 39)
		50	6% (4)	(0, 13)	12% (5)	(1, 22)
		60	4% (2)	(0, 7)	7% (3)	(2, 13)
		70	2% (1)	(0, 4)	4% (2)	(1, 8)
		80	1% (1)	(0, 3)	3% (1)	(0, 5)
	3 years	40	49% (23)	(4, 93)	71% (18)	(36, 100)
		50	34% (15)	(3, 64)	54% (13)	(28, 80)
		60	22% (10)	(3, 41)	38% (8)	(22, 55)
		70	14% (6)	(1, 26)	25% (6)	(13, 38)
		80	8% (4)	(0, 17)	16% (6)	(4, 28)
	5 years	40	71% (23)	(26, 100)	88% (12)	(64, 100)
		50	54% (19)	(16, 92)	76% (13)	(51, 100)
		60	38% (14)	(10, 66)	60% (10)	(40, 80)
		70	25% (10)	(5, 46)	43% (9)	(25, 61)
		80	16% (8)	(0, 32)	29% (10)	(10, 48)
	10 years	40	89% (14)	(62, 100)	97% (5)	(88, 100)
		50	78% (17)	(45, 100)	92% (7)	(77, 100)
		60	62% (17)	(30, 95)	82% (9)	(65, 100)
		70	45% (15)	(16, 75)	68% (10)	(48, 88)
		80	31% (13)	(5, 57)	51% (13)	(25, 77)
	20 years	40	97% (6)	(85, 100)	99% (2)	(96, 100)
		50	91% (10)	(71, 100)	98% (3)	(91, 100)
		60	81% (13)	(54, 100)	94% (5)	(83, 100)
		70	66% (15)	(36, 96)	85% (8)	(69, 100)
		80	49% (17)	(16, 82)	71% (13)	(46, 96)
LF	1 year	40	1% (0)	(0, 2)	1% (1)	(0, 3)
		50	0% (0)	(0, 1)	1% (1)	(0, 2)
		60	0% (0)	(0, 0)	0% (0)	(0, 1)
		70	0% (0)	(0, 0)	0% (0)	(0, 1)
		80	0% (0)	(0, 0)	0% (0)	(0, 0)
	3 years	40	4% (3)	(0, 10)	9% (4)	(1, 17)
		50	3% (1)	(0, 5)	5% (2)	(1, 9)
		60	2% (1)	(1, 3)	3% (1)	(1, 5)
		70	1% (1)	(0, 2)	2% (1)	(0, 3)
		80	1% (0)	(0, 1)	1% (1)	(0, 2)
	5 years	40	8% (5)	(0, 19)	16% (7)	(2, 30)
		50	5% (3)	(0, 10)	10% (3)	(3, 17)
		60	3% (2)	(0, 6)	6% (2)	(2, 10)
		70	2% (1)	(0, 4)	4% (1)	(1, 6)
		80	1% (1)	(0, 2)	2% (1)	(0, 4)
	10 years	40	18% (10)	(0, 37)	32% (12)	(7, 56)
		50	11% (5)	(0, 21)	21% (7)	(8, 33)
		60	7% (3)	(0, 13)	13% (4)	(5, 20)
		70	4% (2)	(0, 8)	8% (3)	(2, 13)
		80	2% (1)	(0, 5)	5% (2)	(0, 9)
	20 years	40	30% (15)	(0, 60)	50% (16)	(18, 82)
		50	19% (9)	(2, 37)	34% (10)	(14, 54)
		60	12% (5)	(2, 22)	22% (7)	(10, 35)
		70	7% (4)	(0, 14)	14% (5)	(4, 24)
		80	4% (3)	(0, 9)	9% (3)	(0, 17)

PNF = percutaneous needle fasciotomy; LF = limited fasciectomy; FDR = first degree relative
