# Supplementary material for: Treatment Durability of Limited Fasciectomy versus Percutaneous Needle Fasciotomy for Dupuytren Disease
Source: Plast Reconstr Surg. 2024 Jan 30;154(5):928–38. doi: 10.1097/PRS.0000000000011322 (PMC11512621; doi:10.1097/PRS.0000000000011322)
Supplement: Supplementary file 4 [file prs-154-0928e-s004.pdf]

Table, Supplemental Digital Content 4. Maximum likelihood estimates and 95% asymptotic confidence interval

Effect of the confounders on the time to first treatment and the time to retreatment using the Wald test. * = statistically significant effect. FDR = first degree relative

Variable	Time to	Estimate (SE)	95% CI	Test statistic	p-value
Gender	first treatment	0.14 (0.19)	(-0.24, 0.51)	0.71	0.48
FDR	first treatment	0.26 (0.17)	(-0.08, 0.60)	1.50	0.13
LF	retreatment	-2.82 (0.37)	(-3.55, -2.09)	-7.56	<0.001*
Age at first treatment	retreatment	-0.47 (0.17)	(-0.81, -0.13)	-2.72	0.007*
Gender	retreatment	0.71 (0.48)	(-0.23, 1.66)	1.48	0.14
FDR	retreatment	0.50 (0.33)	(-0.14, 1.14)	1.54	0.12
